# On the Sense of Equilibrium, and on Its Disturbance

**Published:** 1834-07-01

**Authors:** Herbert Mayo

**Affiliations:** Professor of Anatomy and Physiology in King's College, London.


### *On the Sense of Equilibrium, and on its Disturbance.* By Herbert Mayo, F.R.S., Professor of Anatomy and Physiology in King's College, London.

Among the diversified investigations which Magendie has so successfully pursued in physiology, there is not one which has furnished more singular and unexpected results than his experimental display of the influence of different parts of the enkephalon in originating impulses to locomotion. The experiments, and their results, are the following.

1. If the hemispheres of the brain are removed by an horizontal section through the striated bodies, the animal exhibits a permanent tendency towards locomotion forwards.

2. If the upper half of the cerebellum be removed, the animal exhibits a permanent tendency towards locomotion in the contrary direction.

3. If one tubercle is deeply wounded, the animal exhibits a permanent tendency to move in a circle, which is shewn equally in swimming and in crawling, in flying and in walking.

4. If the pedicles of the cerebellum, or the lateral part of the cerebellum, be cut through vertically on one side, the animal exhibits a permanent tendency to roll over and over towards that side.

5. When one of these experiments has been made, and the tendency to locomotion in a definite direction has been developed, if the counter-experiment, or the injury which determines the opposite motion, be made, the animal stops; the one tendency balances or extinguishes the other.

Magendie somewhere concludes, from these experiments, that the voluntary frame in the healthy living body is subject to, or naturally placed under, different impulses, the opposition of which causes an equilibrium, that is disturbed on the abstraction of one of the component forces. It forms, however, a more probable solution of the phenomena to suppose that the impulses to locomotion have no existence antecedently to the lesions of the brain, but that they originate in, and are caused by, the organic injury; that, for instance, a mechanical injury of one tubercle produces vertigo, or the sense of turning or swimming in the head, to which sensation, when at a certain degree of aggravation, the animal yields, following the whirl of its feelings by its bodily movements; and that injuries of the other parts of the brain produce sensations analogous to vertigo, such as that of moving forwards, backwards, or rolling over, and these again with sufficient intensity to force the voluntary efforts of the animal to follow.

The experiments of Magendie, which have been referred to, have therefore a pathological rather than a physiological bearing; they render it easy to comprehend how an altered condition of the brain may disturb the sense of equilibrium, but they do not explain upon what that sense is founded. The true explanation of this feeling was given by Darwin: his theory, which I have adopted, is to be found in the first volume of the Zoonomia; and the remarks which I have to offer upon the subject contain little that is original, although they may be new to the reader. I think, however, that I have added something to the notion of a muscular sense, which Darwin, as far as I am acquainted with his writings, rather assumed the existence of, than properly understood. The most that has hitherto been known upon this subject was made out by Dr. Wells; after him, by Dr. Spurzheim.

Our sense of support, or steadiness, or equilibrium, is not derived from a single organ of sensation, but is the resultant of impressions made upon three senses, which contribute, however, in very different degrees to its production. These senses are the muscular sense, sight, and hearing.

1. *Of the Muscular Sense.* The most appropriate term for this sense is the sense of effort. Every muscular action is attended with a sensation, which is a measure of the exertion made.

The ideas which the muscular sense communicates are the following:

A. In common with every other sense, it suggests the idea of locality.

B. In common, again, with the other senses, it suggests the idea of something external, as the cause of the sensations experienced.

C. The sense of effort made, or of resistance encountered, gives origin to the idea of force.

D. One set of impressions on the muscular sense gives origin to the idea of different kinds of resistance in the composition of masses of matter, of their hardness or softness, of their elasticity or non-elasticity.

E. Other modifications of muscular sensation convey ideas of the resistance of masses, whether they be fixed or movable, and in what degree.

F. With the preceding impressions is combined a sense of the direction in which the effort is made, or in which resistance is encountered.

The muscular sense has its seat in branches of the same nerves (the ganglionic portions of the fifth and spinal nerves,) which furnish sentient filaments to the integuments. It is, therefore, not surprising that the living muscle feels heat and cold, as the skin does, although perhaps not in the same degree. The sense of effort, on the contrary, is the peculiar endowment of the muscular branches; as the sensations by which we feel roughness, smoothness, and the like, are exclusively excited upon the branches distributed to the skin.

But there is another sense to which it is presumable that both sets of branches, the cutaneous as well as the muscular, minister. This sense is the sense of passive motion, or of its contrary, passive rest, rest without effort. Whenever the inertia of the body is disturbed a sensation is excited. When the frame is passively supported, as in lying down aboard ship, or in a carriage, we are sensible of the change from motion to quiescence, or the reverse, of changes in the rate of motion, and even of the direction of motion, whether it be upwards, downwards, forwards, backwards, sideways, or rotatory. In these instances, however, it is the idea communicated, which alone is simple, not its cause. At any change it is evident that there will be an impression upon the surface of the body, and a vague muscular effort excited likewise; and our feeling of the rate and direction of motion must result from the agitation of all the parts of the frame which are endowed with any modification of touch, or rather, from the combined impressions made upon them all.

The sensation of passive support is the sense of the absence of motion, conjoined with the absence of muscular effort: it bears the same relation to the sensations that have been previously described, which the sense of darkness bears to sensations of colour.

A person who is blind and deaf must preserve his equilibrium through the means of sensations of muscular effort and of touch alone. When he rises from a state of passive support, or of complete bodily repose, he lifts up, by the successive flexions and extensions of his joints, segment after segment of his frame, feeling by his muscular sense the exertions necessary to sustain and balance his weight; and at each change of posture, or even in continuing to maintain the same posture, and at each instant, the guiding sense is used to measure at once and to call for the change or renewal of the effort.

One who, through being born blind and deaf, has been compelled to learn to preserve his equilibrium through the muscular sense and touch alone, moves and supports himself with perfect steadiness and precision, resting upon these senses. But those who have no such original defect employ other senses, especially that of sight, to assist in measuring their steadinesss or unsteadiness of posture. They not merely feel the muscles pulling on different aspects of the joints, and the resistance they have to encounter, and are not merely conscious of holding up the frame by muscular efforts, in the same manner as in other positions they cling by the hands and feet to prevent falling, but they likewise lean upon their sight, or upon their eyes, as upon crutches, feeling steadily balanced, when each visual object around maintains a visually fixed position.

II. *Of Vision.* The law of vision, which is *principally* made use of in conveying the impressions which guide us in maintaining an equilibrium, is the law of visual direction. Through it we have definite knowledge of the direction of each point in space that we see. The law may be thus expressed. The retina is so constituted, that, when any point of it is excited so as to see, the object or the spectrum appears to be situated vertically opposite to the point of the retina so excited. This endowment of the retina is simple, and independent of any motion, or sense of motion, in the muscular apparatus of the eye. The beautiful experiments* through which this law was discovered were made by Scheiner. The following ruder experiment was thought of by myself, which, if the law as above stated were correct, should have a result so definite and simple as at once to prove and to explain the law without recourse

* See "Outlines of Physiology," by HERBERT MAYO, F.R.S. &c.

to the elaborate evidence contrived by Scheiner. When, by making pressure upon the globe of the eye, the retina is disturbed, it is well known that a coloured spectrum is produced. I found that, upon making pressure on different parts of the eye, the place of the spectrum obeys, or is determined, in every instance, by the law of visual direction above enunciated, appearing always to be vertically opposite to the point of the retina that is compressed.

Through this law of visual direction, which enables us to measure exactly our relative position to other objects, we acquire more rapidly than we otherwise should do, and more perfectly, the means of judging of and maintaining the bodily equilibrium. But, at the same time, we exercise all the less the muscular sense; and, when it becomes necessary to resort to that alone as our guide, we sensibly feel its imperfectness, from the contrast of our present state with the certainty of sensation which we before enjoyed. In the dark, for instance, or with the eyes closed, we are unable to walk in a straight line; and, in attempting it, feel a strain and effort of attention to be necessary to do that imperfectly which habit has rendered easy to the blind. A case which bears a curious analogy to this is the difference of the power of distinguishing distances, which a person long blind of one eye exhibits, from one who, having perfect vision with both, for experiment's sake, looks with one only. The former has a vast superiority; for the latter has been in the habit of attending to and calculating upon the convergence of the optic axes, as the object of vision is nearer or more remote, which motion forms no part in the vision of the other.

The influence of sight in maintaining the sense of equilibrium is even better exemplified by disturbing visual impressions than by wholly excluding them. A person who, without previous practice, looks upon a mirror swinging to and fro, or upon the ripple of an extensive sheet of water, or down a steep declivity, becomes giddy: he has no longer a sense of steadiness, or of equilibrium. The visual impressions on which he habitually rests now are uncertain: the objects upon which his eyes used to dwell are either in motion, or at an uncertain distance; there is a relative unsteadiness, and whether it be in the object or in himself he cannot determine, and becomes giddy.

Habit enables one to disregard these uncertain impressions, and by confining the attention to the muscular sense, to preserve the sense of equilibrium on shipboard, or amid alpine scenery. Bodily debility, on the other hand, weakens our reliance upon our senses, and makes us doubly susceptible of giddiness on the kind of occasions which are here adverted to.

It is perhaps in the state of nervous debility alone that the influence of hearing, in maintaining or disturbing the sense of equilibrium, can be observed. At such a time, when loud and confused sounds strike upon the ear, the frame is perceptibly less steady, and the patient nervously looks around for external support.

The distress produced by removing the support of one sense is greatly aggravated by taking away that derived from a second. Such is the distress at first commonly experienced in a swing, or on board ship.

As practice will do so much in enabling one to preserve his steadiness, while all around is moving, and when for each second of time there must be a fresh adaptation of the frame to its shifting position, so is there a prodigious natural diversity among men in this respect. Some instinctively and at once adapt themselves, like boys placed on horseback, pliantly and easily to each variation of position. In others the body is unmanageable and unadaptive, and giddiness is immediately produced. I have generally remarked, that those who, without practice, can bear the motion of a swing or of a vessel, bear with equal freedom from uneasiness the looking down from great elevations.

The *continuance of giddiness* that has been produced by the motion of a vessel, or by turning round many times in succession, *after the cause has ceased*, deserves to be examined. It is referable to a principle of universal operation through all the mental actions. There is a law of mental induction, which extends the effects of an impression to some period after it has ceased. The anatomist who has been busied all day in teazing out filaments of nerves, when he closes his eyes to rest, is perplexed by the entanglement of threads, which his vivid conceptions force upon him. In the same manner, in one of the instances before us, the strong conception of turning remains for a long time after the body has been stopped, and we feel no little tendency to follow the conception, and realize the motion.

Giddiness strongly excited, either by continued turning or by the motion of a swing, or of a vessel, leads to nausea and vomiting. Dr. Wollaston referred the effect in some of these instances to the altered momentum of the blood in the brain. I believe that they admit of another, and a more general and a more just explanation. Giddiness, nausea, and vomiting, appear to me the natural consequences of the disturbed sense of equilibrium: there appears to be the same alliance between this sense and the effect on the stomach, as between some of the other senses (taste, for instance, or smell,) and the same. Disturb the sense of equilibrium, or present any extremely nauseous effluvia to the nose, and in either case vomiting follows.

Those who are very susceptible of disturbance of the sense of equilibrium know that one of the different causes which most excite distress and nausea, is the feeling of sinking or descending from want of support; as, for instance, when one is in a vessel that is pitching, or when a person is seated in a carriage with his back to the horses. The sense of being lifted up, on the other hand, is a strong relief, after the distress attending the sensation of failing support. As far as I can see, this difference is entirely arbitrary.

The following argument appears to me conclusive in favour of the hypothesis, that nausea, in these instances, is directly dependent upon disturbance of the sense of equilibrium. When a person has taken wine too freely, if he closes his eyes, a sensation of vertigo arises, accompanied with nausea. The vertigo no doubt results from some affection of the brain, analogous, in its temporary effects at least, to the injury of the tubercles in Magendie's experiments. Now, if the patient, under these circumstances, opens his eyes, and fixes them upon any distinct and stationary object, the internal source of whirling appears arrested, and the sense of equilibrium is quickly restored. At the same time the nausea goes off,—so closely and directly is it connected with the sense of disturbed equilibrium. On the recommencement of the inward whirl, produced by again closing the eyelids, the nausea returns; on steadying by the eye the whirling sense, the nausea again disappears.

